# Validation of a Novel Electronic Health Record Patient Portal Advance Care Planning Delivery System

**DOI:** 10.2196/jmir.9203

**Published:** 2018-06-26

**Authors:** Seuli Bose-Brill, Michelle Feeney, Laura Prater, Laura Miles, Angela Corbett, Stephen Koesters

**Affiliations:** ^1^ Division of General Internal Medicine, Department of Internal Medicine College of Medicine The Ohio State University Columbus, OH United States; ^2^ Medical Student Research Program College of Medicine The Ohio State University Columbus, OH United States; ^3^ College of Public Health The Ohio State University Columbus, OH United States

**Keywords:** advance care planning, electronic health records, patient portal

## Abstract

**Background:**

Advance care planning allows patients to articulate their future care preferences should they no longer be able to make decisions on their own. Early advance care planning in outpatient settings provides benefits such as less aggressive care and fewer hospitalizations, yet it is underutilized due to barriers such as provider time constraints and communication complexity. Novel methods, such as patient portals, provide a unique opportunity to conduct advance care planning previsit planning for outpatient care. This follow-up to our pilot study aimed to conduct pragmatic testing of a novel electronic health record-tethered framework and its effects on advance care planning delivery in a real-world primary care setting.

**Objective:**

Our intervention tested a previsit advance care planning workflow centered around a framework sent via secure electronic health record-linked patient portal in a real-world clinical setting. The primary objective of this study was to determine its impact on frequency and quality of advance care planning documentation.

**Methods:**

We conducted a pragmatic trial including 2 sister clinical sites, one site implementing the intervention and the other continuing standard care. A total of 419 patients aged between 50 and 93 years with active portal accounts received intervention (n=200) or standard care (n=219). Chart review analyzed the presence of advance care planning and its quality and was graded with previously established scoring criteria based on advance care planning best practice guidelines from multiple nations.

**Results:**

A total of 19.5% (39/200) of patients who received previsit planning responded to the framework. We found that the intervention site had statistically significant improvement in new advance care planning documentation rates (*P*<.01) and quality (*P*<.01) among all eligible patients. Advance care planning documentation rates increased by 105% (19/39 to 39/39) and quality improved among all patients who engaged in the previsit planning framework (n=39). Among eligible patients aged between 50 and 60 years at the intervention site, advance care planning documentation rates increased by 37% (27/96 to 37/96). Advance care planning documentation rates increased 34% among high users (27/67 to 36/67).

**Conclusions:**

Advance care planning previsit planning using a secure electronic health record-supported patient portal framework yielded improvement in the presence of advance care planning documentation, with highest improvement in active patient portal users and patients aged between 50 and 60 years. Targeted previsit patient portal advance care planning delivery in these populations can potentially improve the quality of care in these populations.

## Introduction

### Background

Advance care planning (ACP) is the formal process of outlining a patient’s future care preferences should they lose the ability to make informed decisions for themselves [1,2]. ACP documentation provides guidance in accordance with patient care preferences to proxy decision makers and medical teams in times of medical crisis. Patients with documented ACP experience increased adherence to their desired medical preferences [3], higher rates of palliative management, fewer hospitalizations, and increased quality of life near death [1]. Similar studies have found that patients with a plan for future medical care spend less time in the hospital during their last year of life and have their wishes more frequently respected by family members [1,4-6]. ACP also has the potential to reduce expensive health care interventions not wanted by the patient, such as lengthy critical care stays at the end of life [7]. Despite these benefits, the Center for Disease Control and Prevention estimates that ACP completion rates are around 30%, even with advancements in the electronic health record (EHR) and ACP delivery [8].

The Institute of Medicine recommends conducting ACP early in a patient’s chronic disease diagnosis, with periodic reassessment every several years or with change in prognosis (such as new diagnosis, hospitalization, or worsening of chronic disease) [9]. This ACP communication is best provided in the primary care setting [10]; yet, primary care remains ill-equipped to systematically conduct ACP discussions due to the competing care demands and fast pace of appointments [11]. As few as 1% of Medicare beneficiaries with an established primary care physician report having an ACP conversation with their health care provider [12]. Identified barriers include provider time constraints, uncertain patient prognosis, emotional complexity of ACP decisions, and difficulty in information sharing within and between health organizations [13]. The Centers for Medicare and Medicaid have recognized the need for early ACP by providing reimbursement for team-based primary care ACP discussions occurring under physician supervision [14]. These current gaps in care, juxtaposed with the urgent need for effective outpatient ACP care models, require development and rapid dissemination of innovative ACP strategies [15].

EHR-linked patient portals, first described in the 1990s and now ubiquitous due to EHR Meaningful Use guidelines [16], allow patients to electronically communicate with their medical providers within a secure platform. Patient portal communication has driven innovation in chronic disease management, population management, and previsit planning with strategies such as incorporating a patient portal refill button for hypertension patients; sharing top-priority problem list information with complex diabetes patients; and providing influenza vaccine outreach. These strategies allow providers to more effectively use time during an appointment by providing preparatory communication to patients before the appointment begins [17-21]. Patient portal–based outreach for health maintenance has been reported to marginally improve health maintenance behaviors such as flu vaccination (by 1-2%) [17]. Response rates to provider-initiated patient portal communication for chronic disease management have been reported at about 15% [22]. Patient portals are not without their own unique barriers; one study found that patients chose not to activate their portal due to a lack of sufficient instructions, privacy concerns, preference for face-to face interaction, or connectivity obstacles [23-26]. Despite these limitations, patients and care teams are increasingly actively incorporating portals into medical management.

### Framework and Objectives

In an earlier study, our research team developed and pilot-tested a concise EHR patient portal–linked, electronic ACP communication framework in a small randomized controlled trial [11,27]. The framework was developed through incorporating best practice guidelines, and then refined through focus group feedback and cognitive interviewing [11]. This developed framework consists of an introduction to ACP and key evidence-based questions that can be sent to patients for response outside of their office visit. By allowing patients to think and comment about their future wishes for care in advance of visits, we hoped to maximize patient-provider time in the office visit for advanced communication and documentation. The framework responses were automatically stored in a patient’s medical record for retrieval by clinical staff or physicians at office visits. Patient responses to the framework could also be sent to the primary care provider for review [11]. Use of this framework in a small pilot study demonstrated improvement in ACP documentation rates and quality [11]. Even though our framework was the first to be piloted in the field [11], its feasibility and impact on outcomes in a real-world primary care setting when integrated into actual previsit planning algorithms in the course of actual clinical primary care were yet to be determined [28].

The aim of this study was to determine the impact of previsit ACP planning using a secure EHR-linked framework upon ACP documentation when incorporated into a real-world primary care environment.

## Methods

This study was approved by the Ohio State University Institutional Review Board.

### Sample

**Figure 1:**
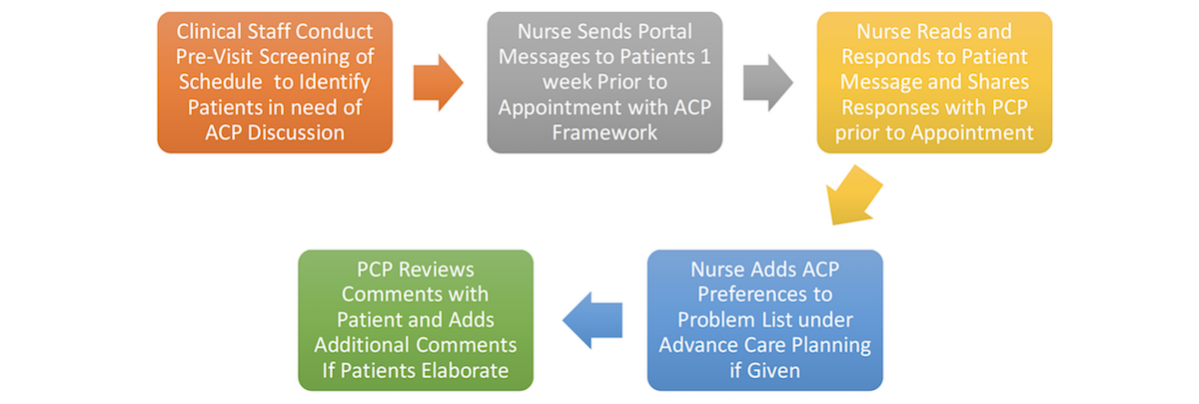
Intervention workflow that was implemented at the study practice during the 3-month trial period. ACP: advance care planning; PCP: primary care physician.

Patients 50 years or older, presenting for a preventive health or chronic disease follow-up visit, with an active MyChart account, at a participating clinical site were included in the study. Patients did not need prior MyChart experience. There were 2 clinical sites participating in the study. Sites were selected based on their demographic, size, and provider similarity, as well as their uniform clinical practices with respect to ACP delivery. Each clinical site used the same ACP practices before the study period, which included an institutional packet of information on ACP, state-issued documents about Advance Directives (ADs), and encouragement to discuss any ACP questions with their provider. The usual care site maintained these practices throughout the duration of the study. The intervention site incorporated an open-ended ACP framework (containing 4 questions), sent via a patient’s EHR-tethered patient portal, into a clinical practice algorithm. Physicians, nurses, and other clinical staff at the intervention clinic were collectively involved in developing this ACP previsit planning algorithm that was rolled out practice wide over a 3 month period (Figure 1). The algorithm focused on promoting patient/provider communication surrounding ACP preferences, rather than intervening specifically on completed ADs scanned into the EHR. Furthermore, 219 patients in the control group and 200 patients in the intervention group were cared for during the study period, yielding 419 total participants. Further demographic characteristics are outlined in Table 1 for both the control and intervention sites. This study measured the impact of a clinical intervention rolled out practice wide. Our study team received a Waiver of Consent by the Institutional Review Board to assess the pragmatic impact of this clinical intervention. It was not possible to blind this study because chart review automatically revealed participants who received the ACP framework versus those who did not. We mitigated the inability to blind this study by using binomial metrics, such as documentation present/absent, and rigid scoring criteria for quality. We also conducted spot checking of reviews to ensure accuracy of dataset, as outlined above.

Participants did not know their intervention was the intervention of interest. Each clinical site agreed to participate in an ACP process study. However, providers and patient participants did not receive labels about whether they were receiving intervention or usual care. Practice workflow was implemented without labels.

Data security was paramount in this study. We used clinical staff (Institutional Review Board approved) routinely interacting with the patient record and completing previsit planning for clinical care to administer the intervention. Our research team was embedded in the clinical site. Data were housed within the secure institutional firewall and only accessed within the clinical site. Only de-identified datasets were shared with the statistical team for analysis using a secure, institutional drive. The delivery system developed by the practice providers had built-in safeguards for addressing clinical emergencies, such as patients responding to the secure message with medical complaints, by having a nurse and physician on call for urgent messages.

### Measures

Charts were reviewed both 1 week and 4 weeks post appointment. Charts were reviewed by a member of the team who had received training and quality control checks on ACP chart review protocol. Chart review findings were spot checked by a second member of the team (one every 20 records) to ensure accuracy. The protocol outlined that training and education interventions would be used to respond to discrepancies in chart review rates and quality assessments. However, interventions were not needed because spot checking did not yield discrepancies. The participant’s demographics, presence of ACP (including before and after the visit), quality of ACP if present, and number of MyChart messages sent in the last year were recorded. The intervention charts were reviewed to see if the patient had read the intervention on their portal and responded to any of the questions.

**Table 1:** Demographic information from both the control and intervention clinic.

Demographic information	Control (n=219)	Intervention (n=200)
Median age, years	61	61
Age range, years	50-93	50-91
Male, n (%)	76 (35)	101 (50.5)
Female, n (%)	143 (65)	99 (49.5)
Number of chronic diseases^a^	4	4
Number of medications^a^	7	6

^a^Median number per patient at each practice site.

Quality of ACP was measured using a 20-point scoring criteria entitled “Criteria for Scoring Quality of ACP Documentation” (Multimedia Appendix 1) that has been used in previous studies [11,27]. The rubric was created by our team after reviewing best practice reports on how to measure ACP quality, such as those from the United Kingdom National Health Service, the Australian Quality Advance Care Planning Board, and the National Hospice and Palliative Care Organization’s “Caring Connections” program [29-31]. This method of scoring the quality of ACP has not been validated, but assigned points for the presence of items in the patient’s ACP. The ability to quantify the quality of our patient’s ACP was a crucial component of the study and gave us more thorough feedback on the utility of the novel framework.

### Analysis

Our study analyzed the documentation rates and quality of ACP across both study sites, especially focusing on new ACP documentation appearing in the EHR. To assess whether or not the increase in new documentation was significant between the 2 sites, a Fisher exact test was used. To analyze quality, a Mann-Whitney test was used to test the significance in new ACP quality between the 2 sites. The data were also analyzed by age and portal usage. Participants were separated into age groups by decade and portal usage was defined as either high or low, with high usage being more than 10 portal messages in 1 year.

## Results

### Intervention

Of the 200 patients who were sent the intervention, 156 read the message (78.0% read rate) on their portal and 39 responded (19.5% response rate) to at least one question in the framework (see Multimedia Appendix 2). Of those who responded to our intervention, 49% (19/39) already had some form of ACP documented in their EHR and 51% (20/39) added ACP to their EHR for the first time, yielding a 105% (19/39 to 39/39) increase in ACP documentation rates. Responders with existing ACP had a mean quality score of 4.94, as compared with a mean score of 4.09 for all documented ACP at the intervention arm during our study period. For respondents without prior documented ACP, the intervention alone yielded a mean quality score of 3.7. Respondents sent a median 11 MyChart messages per year and had a median age of 63 years. MyChart usage did not increase due to the intervention at the site overall; patients had a median 5 messages per year at our intervention arm before and during the study period.

**Table 2:** Documented advance care planning (ACP) in electronic health record. Documentation rates represent the percentage of charts that had any form of ACP, and quality is rated by the 20-point scoring criteria.

Patient characteristic			Control	Intervention
**All patients**				
	**ACP^a^ documentation rate, N**		219	200
		Preintervention, n (%)	129 (58.9)	74 (37.0)
		Postintervention, n (%)	130 (59.3)	94 (47.0)
		Rate percentage increase, n (%)	0.7	27.0
	**Quality of all documented ACP, N**		130	94
		Quality rating postintervention, mean	3.26	4.09
**Patients aged 50-60 years**				
	**ACP documentation rate, N**		109	96
		Preintervention, n (%)	54 (49.5)	27 (28)
		Postintervention, n (%)	55 (50.4)	37 (39)
		Rate percentage increase, n (%)	1.8	37
	**Quality of all documented ACP, N**		55	37
		Quality rating postintervention, mean	2.81	3.75
**Patients who are high portal users (>10 messages in 1 year)**				
	**ACP documentation rate, N**		82	67
		Preintervention, n (%)	51 (62)	27 (40)
		Postintervention, n (%)	51 (62)	36 (54)
		Rate percentage increase, n (%)	0	33
	**Quality of ACP documentation, N**		51	36
		Quality rating postintervention, mean	3.25	4.19

^a^ACP: advance care planning.

Our intervention did not appear to affect the percentage of patients who had a scanned document in their EHR; both before and after the intervention, approximately 14% (28/200 and 7/47) of patients had a scanned directive at that practice. One patient brought in an Advance Directive to be scanned after responding to our framework.

### Documentation and Quality Rates

ACP documentation in the EHR increased by 27.0% (74/200 to 94/200) during the study period at our intervention site, compared with a 0.7% (129/219 to 130/219) increase at our control site (Table 2; see Multimedia Appendix 1 for scoring criteria used for rating quality). A Fisher exact test was used to determine the significance of the differing increase in new documentation rates during the study period and yielded *P*<.001, indicating that patients exposed to our intervention were more likely to document ACP than those receiving usual care. A Mann-Whitney test was used to see if new ACP documented under our intervention was higher in quality and yielded *P*<.001, indicating that having the intervention led to a statistically significant ACP quality difference.

### Age

Patients aged between 50 and 60 years saw the greatest increase in ACP completion rates. At our intervention site, documentation rose 37% (27/96 to 37/96) as compared with 1.8% (54/109 to 55/109) at the control site. Comparatively, the 61-70 age group saw a 31% (29/76 to 38/76) increase in documentation rates, and the 71-80 age group saw a 6% (17/26 to 18/26) increase at the intervention site. Our control site had a 0% increase in each of those 2 age groups, but higher baseline rates of ACP completion before the study period (64% and 81%). In the intervention arm, there was only 1 patient between 80 and 89 years, and 1 patient over 90 years, so there were insufficient data to analyze this group. Individuals in the 50-60 age group, however, had slightly lower ACP quality as compared with the study population as a whole.

### MyChart Users

Those who sent more than 10 MyChart messages in 1 year were defined as “High Portal Users” and comprised approximately a third of the study group at each site. Documentation rose by over 33% (27/67 to 36/67) at the intervention site for this group. Comparatively, low portal users (10 messages or less) at our intervention site saw a 23.4% (47/133 to 58/133) increase in documentation rates.

## Discussion

### Patient Applications

In this study, we found that patients exposed to our framework were significantly more likely to have ACP documentation in the EHR and the quality of that documentation was better. This intervention benefits both the patient and the provider by providing another way for patients to think through the difficult decisions of how they envision their future care before their office visits. For patients who already used the patient portal, adding the framework would be a seamless integration into their usual part of their care. This tool was used most frequently by patients in the 50-60 age group and already active on MyChart. Targeting patients who are high users or in this age demographic to receive this tool can be a strategy for providing high-yield individualized previsit planning for ACP using patient portals. This intervention did not capture many different demographics, including all nonportal users, so developing other strategies to improve ACP documentation against cultural, technological, and demographic barriers must be used to ensure that there are improved outcomes for all and to continue to address existing health disparities in ACP documentation [32]. Previous research has found that patients who are middle-aged, male, and have greater disease burden are more likely to use their patient portals, which is similar to the trend we found in our study [33]. Few studies report on response rates to practice level, provider-initiated patient portal interventions; however, response rates to our framework (20%) using this previsit planning system were higher than response rates to a similar intervention for primary care depression screening and management (15.4%) using a secure patient portal messaging system [22].

### Office Workflow Applications

In terms of workflow, the framework requires a member of the care team to send out the MyChart message 3-5 days before the appointment. If a patient responded, answers were appropriately documented and sent to the patient’s provider. In our study, the messages were sent out by a clinic nurse who could also answer any follow-up questions the patient had and then route the message to the appropriate provider. With increased team-based, patient-centered medical home patient outreach before appointments in primary care settings, as well as ubiquitous use of patient portals for practices to adhere to meaningful use guidelines, these interventions can be disseminated to a wide array of primary care practices. Higher rates of ACP documentation resulted, while reducing time needed to have a complete ACP discussion with the patient during the office visit, as existing answers have already been recorded and the patient had preparatory time to articulate their wishes.

### Long-Term Clinical Applications

Previous studies have shown the benefits of previsit planning; if the provider has documentation of some of the patient’s future care preferences beforehand, there can be a more productive discussion with the patient during their appointment [34]. As end-of-life discussions can be difficult for patients, having a standardized framework that is sent to all patients over 50 years helps normalize the discussion and better prepare the patient [34]. Physicians can also tailor their future ACP discussions based off the patient’s documented answers from the framework, allowing them to be more effective conducting individualized ACP discussions [35]. For provider workflow, having the ACP process begin before the appointment can save time during the appointment and documentation afterwards, helping to ensure communication about end-of-life preferences occurs despite competing priorities [36].

The study was not designed to elicit qualitative feedback from patients and providers to promote its pragmatic implementation. However, the participating site liked the delivery system enough to implement it as a permanent intervention. Furthermore, the participating site shared the intervention, which has now been disseminated to the wider net of associated primary care sites at the institution.

### Limitations

Baseline rates of ACP documentation at each site were different, as noted in the results section, with the usual care site having higher rates of completed ACP documentation at baseline. However, preintervention chart review at both sites allowed assessment of typical documentation rates, to determine the change in documentation rates in intervention versus control before and after the intervention period. Additionally, patients had to have an activated MyChart account to be included, which excluded a portion of the clinic population.

### Conclusions

Incorporating the patient portal into ACP delivery is a promising way to increase completion rates and efficiently facilitate the conversation between the provider and the patient about their future wishes. This strategy may be more effective in patients familiar with patient portal use, who regularly use patient portal communication to access clinical care.

## Appendix

Multimedia Appendix 1 Advance Care Planning Quality Grading. Criteria for scoring quality of ACP documentation in the EHR.

Multimedia Appendix 2 Framework Questionnaire.

## Abbreviations

ACP: advance care planning
EHR: electronic health record
