# Supplementary material for: Validation of a Novel Electronic Health Record Patient Portal Advance Care Planning Delivery System
Source: J Med Internet Res. 2018 Jun 26;20(6):e208. doi: 10.2196/jmir.9203 (PMC6039766; doi:10.2196/jmir.9203)
Supplement: Multimedia Appendix 1 [file jmir_v20i6e208_app1.pdf]

Appendix A

Advance Care Planning Quality Grading

<u>Criteria for scoring quality of ACP documentation in the EHR (Developed after review of Caring Connections, Australian State of Science on Quality Advance Care Planning)</u>

Exploration of:

-Did you assess if patient can make own decisions (1 point)

-Did you look at why patient is completing ACP document (1 point)

-Does the patient understand the following:

- Their illness? (1 point)
- Why they are doing ACP? (1 point)
- All the different ways they can document their future plans? (1 point)
- When documented preferences would be activated (when they are unable to voice their preferences)? (1 point)
- That the ACP document is not fixed in stone and can be changed as their wishes change? (1 point)

-Patient values and circumstances:

- Does the patient have any cultural or religious preferences? (1 point)
- Does the patient have any past experiences with ACP? (1 point)
- Does the patient report any barriers to care? (1 point)

-Picking a decision maker: Did the documentation record:

- Who would serve as a healthcare agent? (1 point)
- Agent's willingness to participate in patient's care? (1 point)
- Agent's understanding of the patient goals, values, and beliefs? (1   point)
- Agent's ability to make decisions when under stress? (1 point)
- Agent's willingness to honor the patient's desires when ACP activated? (1 point)
- Agent's participation in the ACP discussion (during visit or with patient)? (1 point)
- How much freedom the patient gives their agent to change his/her voiced preferences? (1 point)

-What does the patient want to accomplish?

- What contributes to a positive quality of life for the patient? (1 point)
- How would the patient envision his/ her care if s/he could not make own decisions, didn't know who s/he was, and/ or where s/he was (clinical situations such as dementia)? (1 point)

- Seeing it through?

  Did the patient get a follow up offer to have additional conversations if desired, including, but not limited to, referrals to sub-specialists, or inter-professional disciplines like social work, chaplaincy, etc answer questions about ACP? (1 point)
