# Supplementary material for: Validation of a Novel Electronic Health Record Patient Portal Advance Care Planning Delivery System
Source: J Med Internet Res. 2018 Jun 26;20(6):e208. doi: 10.2196/jmir.9203 (PMC6039766; doi:10.2196/jmir.9203)
Supplement: Multimedia Appendix 2 [file jmir_v20i6e208_app2.pdf]

Appendix B
Framework Questionnaire

Dear (*patient name),*

This is (*clinic nurse)*. Please complete the following questions if you have time before your next office visit.

As a routine part of your healthcare, we would like you to answer some questions. These questions help us know how we should take care of you in case you ever get too sick to speak for yourself. Your answers will be filed into your chart to help guide your future care. You can change your answers at any time.

Please think about these questions and talk about them with your loved ones. Send us back your answers when you are done.

If you are too sick to speak for yourself, who should make medical decisions for you?

Is there anyone you specifically would NOT want to make medical decisions for you?

What activities give your life quality?

What helps you cope during hard times (like the death of a loved one or a serious illness)?

If you would like to discuss your answers more with your physician during an appointment, please let us know. Our office has more resources to help you put your wishes in writing. Ask us for more information the next time you visit the clinic.

Thank you for your answers.

Sincerely, (*clinic nurse)*
